# Value of a lateral inferior pedicle flap in Draf IIb for recurrent frontal sinus diseases: a prospective study

**DOI:** 10.1007/s00405-022-07302-0

**Published:** 2022-02-26

**Authors:** Chao He, Hong-Tao Zhen

**Affiliations:** Department of Otolaryngology-Head and Neck Surgery, Tongji Hospital, Tongji Medical College, Huazhong University of Science and Technology, No. 1095 Jiefang Avenue, Wuhan, 430030 People’s Republic of China

**Keywords:** Endoscopic sinus surgery, Frontal sinus, Draf IIb, Restenosis, Lateral inferior pedicle flap

## Abstract

**Purpose:**

The Draf IIb procedure allows the widest unilateral access to the frontal sinus in a minimally invasive fashion, with efficiency and safety comparable to the Draf III. However, this technique is still associated with a high postoperative stenosis rate. The exposure of drilled bone induces osteitis predisposing to scarring and neo-osteogenesis causing ostium restenosis. We developed a novel lateral inferior pedicle flap (LIPF) to cover the exposed bone and prevent restenosis during Draf IIb. We aimed to describe our technique.

**Methods:**

Adult patients requiring a Draf IIb for unilateral recurrent frontal sinus disease were prospectively enrolled. A LIPF technique was systematically performed. Demographics and complications were recorded. The primary outcome measure was neo-ostium patency at 12 months. In patients with chronic rhinosinusitis (CRS), the clinical control rate was evaluated at 12 months.

**Results:**

59 patients underwent the Draf IIb with LIPF technique from 2013 to 2021. 49 patients (20 women/29 men, median age of 48.0 years) completed at least 12 months of follow-up (median 41.0 months, range 12–100 months). Indications included recalcitrant CRS (*n* = 32), inverted papilloma (*n* = 9) and frontal mucocele (*n* = 8). Overall, the neo-ostium remained patent at 12 months in all patients, and the clinical control rate of 32 patients with recalcitrant CRS at 12 months was 100%. No main complications were recorded.

**Conclusion:**

The LIPF technique was associated with a high rate of success for a Draf IIb.

## Introduction

Despite the major progress in endoscopic approaches, instrumentations and image guidance systems, the endoscopic surgical management of the frontal sinus remains a challenge to surgeons due to the numerous anatomic variations, difficult surgical access, and tendency of restenosis during the follow-up period [1, 2].

Extended frontal approaches, as described by Draf, Gross, and Lothrop, can often improve long-term frontal sinus patency and surgical outcomes [3, 4]. For advanced frontal sinus pathologies (recalcitrant chronic frontal sinusitis, mucocele, cerebrospinal fluid leak, inverted papilloma, and others), the two main endoscopic surgical options are the Draf IIb and Draf III procedures.

The Draf IIb procedure allows the widest unilateral access to the frontal sinus by extending the resection medially to the middle turbinate toward the nasal septum. The wide approach to the frontal sinus is obtained by drilling the frontal process of the maxilla laterally and the nasofrontal beak anteriorly [5]. The Draf III procedure is a bilateral Draf IIb with a superior and inter-sinus septectomy [6]. At present, reports on Draf IIb procedures are scarce, because the Draf III procedure is most often favored, although it is more aggressive, with the solid evidence on its safety and efficacy [7, 8]. However, recent studies have confirmed the efficiency and safety of Draf IIb procedure comparable to the Draf III [9, 10].

The Draf IIb and III procedures have solved the previously mentioned problems by providing better visualization and approach to the frontal sinus and to maximize the size of the created neo-ostium of the frontal sinus, but some limitations remain [3, 4, 7]. Postoperative restenosis after the Draf procedure remains a major problem. The exposure of drilled bone in the Draf IIb and III procedure tends to induce osteitis predisposing to scarring and neo-osteogenesis which causes ostium restenosis [11–14]. To speed up mucosal healing and prevent osteitis, mucosa flaps can be used to cover exposed bone [15]. Several mucosal flaps applied to Draf procedures were reported varied from free grafts to pedicled flaps [2, 5, 11, 16–20]. A free graft is easy to manipulate but is lacking in viability. A pedicled flap is robust and difficult to manipulate, and usually disturbs the operative field [2].

We describe a modification of the Draf IIb procedure for unilateral recurrent frontal sinus diseases, in which a lateral inferior pedicle flap (LIPF) is used to potentially decrease postoperative ostium stenosis. The main objective of the present study was to prospectively evaluate postoperative stenosis regarding this novel technique.

## Patients and methods

### Patients’ selection

**Fig. 1:**
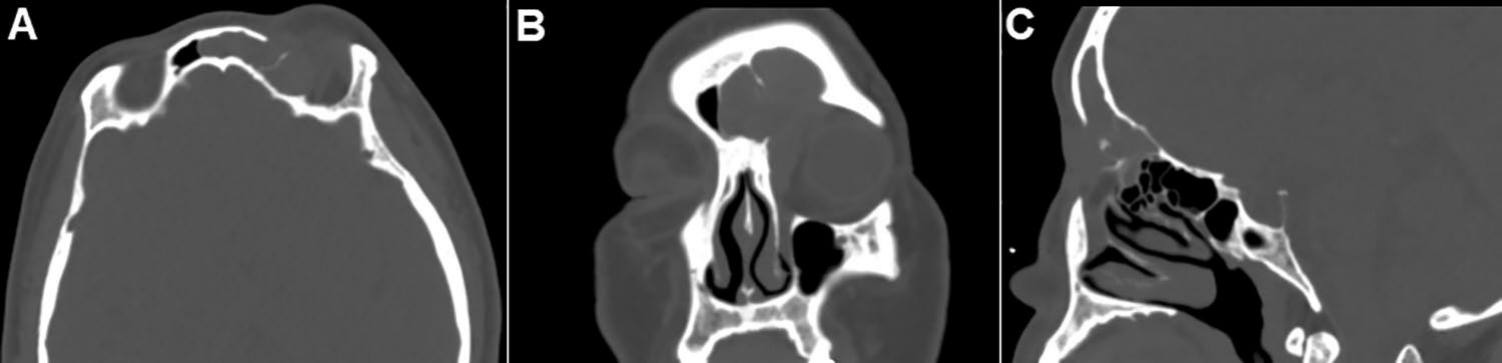
(A) Axial, (B) coronal and (C) sagittal paranasal sinus CT images of a patient with recurrent frontal sinus inverted papilloma who suffered by persistent headache

This is a prospective study approved by the Ethical Committee of Tongji Hospital, Tongji Medical College, Huazhong University of Science and Technology. Adult patients (> 18 years) undergoing the Draf IIb procedure for recurrent frontal sinus diseases (2013–2021) were enrolled. All patients had been treated with at least one previous endoscopic surgery. Diagnosis was confirmed by symptoms and high-resolution CT scans (Fig. 1). During the study period, a LIPF technique was systematically performed unless a technical limitation was encountered or in the presence of an unhealthy flap mucosa due to severe polyposis or fibrosis. Demographics and indications for surgery were collected. Patients were sorted by operation time.

### Surgical technique

Procedures were performed under general anesthesia with orotracheal intubation. Mucosal decongestion was obtained with cotton wool soaked with 0.05% xylometazoline solution. The image guidance (Medtronic Navigation, Inc) was used.

**Fig. 2:**
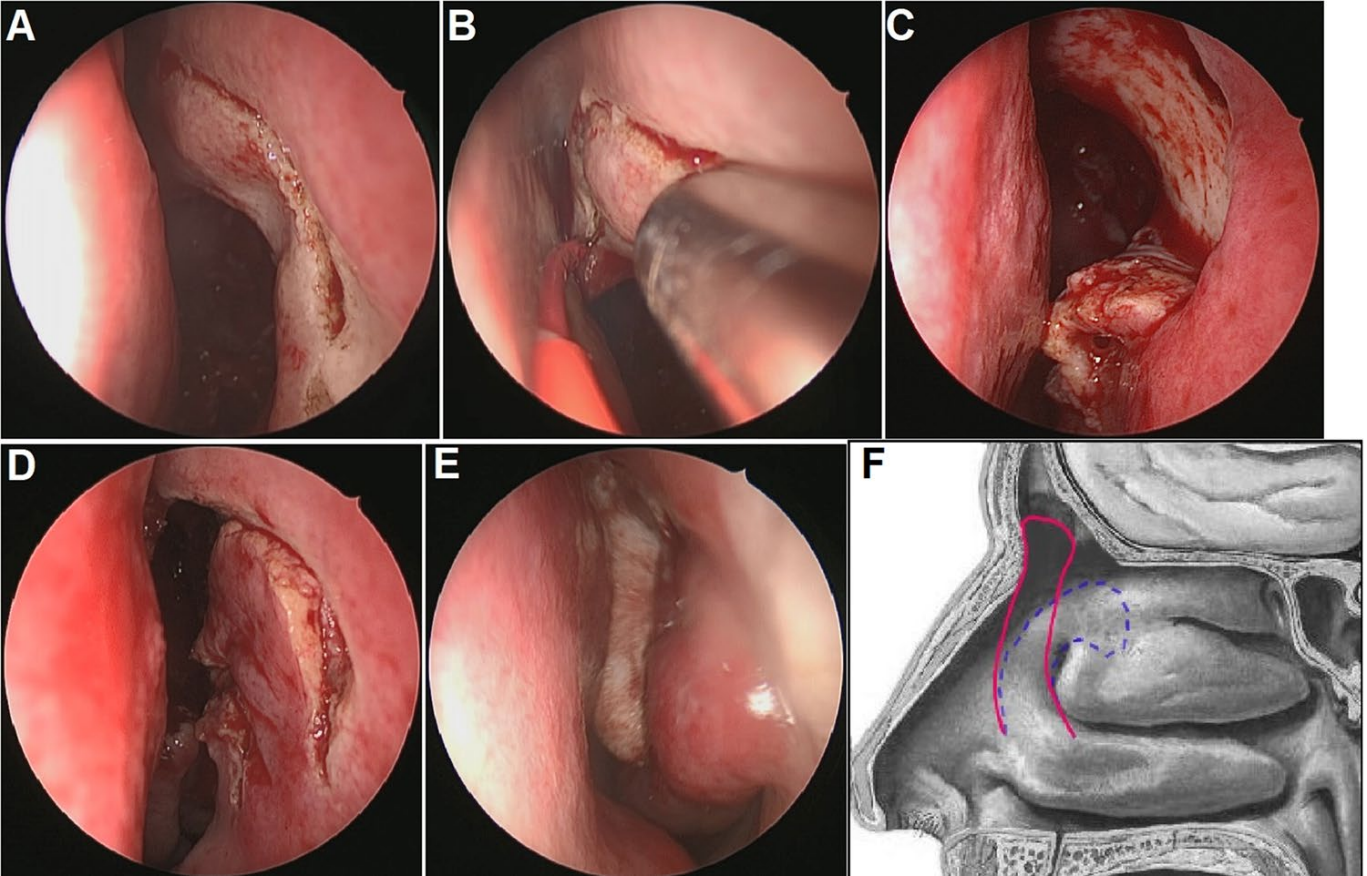
Intraoperative and postoperative endoscopic views of the LIPF technique in the left nasal cavity. (A) A vertical incision was made approximately 10 mm anterior to the axilla of the middle turbinate. (B) This incision ran around the agger nasi and across the axilla. (C) The flap was elevated towards the dorsum of the inferior turbinate. (D) The flap was unfolded to cover the exposed bone. (E) A nasopore was used to stabilize the flap. (F) A schema of the flap is shown. The blue dotted line shows the incision, and the red solid line shows the flap covering the exposed bone

A LIPF was raised after a standard uncinectomy and ethmoidectomy. A vertical incision was made approximately 10 mm anterior to the axilla of the middle turbinate, extending downward to the dorsum of the inferior turbinate parallel to the maxillary line and upward to the roof of nasal cavity (Fig. 2A). Then, from the upper extremity, this incision ran around the agger nasi, across the axilla (Fig. 2B), and downward following the maxillary line. It was worth noting that, for the cases with adequate and healthy middle turbinate, this incision should extend to the medial surface of the middle turbinate to retain as much of the medial mucosa of the middle turbinate as possible to ensure the length of the flap. Meanwhile, for the cases without sufficient available middle turbinate mucosa, free mucosal graft was used as supplement. The flap was elevated in a subperiosteal plane toward the dorsum of the inferior turbinate and then folded and placed in the inferior part of the nasal cavity during the subsequent steps (Fig. 2C). Elevation of this flap was followed by a standard Draf IIb procedure. A wide neo-ostium more than 5 mm was obtained by resection of the floor of the frontal sinus between the lamina papyracea laterally and the nasal septum medially ahead of the ventral margin of the olfactory fossa. The top and anterior walls of the frontal sinus were exposed by drilling the frontal process of the maxilla laterally and the nasofrontal beak anteriorly. At the end of the surgery, the LIPF was unfolded to cover as much of the exposed bone as possible (Fig. 2D). A nasopore was used to stabilize the flap (Fig. 2E). A schema of the flap was shown in Fig. 2F.

### Follow-up and outcome measures

All patients were instructed to perform nasal saline irrigation three times a day for 1 month. For patients with recalcitrant chronic rhinosinusitis (CRS), nasal nebulization inhalation of budesonide (1 ml = 1 mg) was applied once a day after the nasal packing removal at 1 weeks and was continued for at least 3 weeks as described in our previous study [21].

Patients were followed-up for at least 12 months. Complications during the operation and follow-up period were recorded.

The primary outcome measure was postoperative neo-ostium patency at a minimum follow-up of 12 months. The ostium patency was defined as the ability to visualize into the frontal sinus by endoscopic examination, and follow-up CT scan was also used to aid in evaluating ostium patency. Failure was defined as complete closure of the frontal cavity or recurrence of disease requiring rescue surgery.

In patients with recalcitrant CRS, the surgical outcome was evaluated with the clinical control rate at 12 months, including evaluation of symptom and endoscopy. Symptom evaluation and endoscopy were conducted at baseline and follow-up. Symptoms including nasal obstruction, rhinorrhea/postnasal drip, facial pain/pressure, hyposmia, and sleep disturbance/fatigue were scored by visual analog scale (VAS) from 0 to 10, with 0 indicating “no complaint whatsoever” and 10 indicating “the worst imaginable complaint” [22]. Endoscopic physical findings were scored according to the Lund-Kennedy scoring system. Clinical control of CRS was categorized as controlled, partly controlled, or uncontrolled, according to EPOS 2012 [22]. Controlled disease is defined as presenting no bothersome symptoms (VAS score ≤ 5), with healthy, or almost healthy mucosa, and no need for systemic medicine to control the disease. Partly controlled patients experience fewer than 2 of the following: nasal blockage; rhinorrhea or postnasal drip; facial pain or pressure; impaired smell; sleep disturbance or fatigue; diseased mucosa on endoscopy; and a need for rescue treatment within the previous 6 months. Uncontrolled disease is defined as having ≥ 3 of the aforementioned conditions, despite rescue treatment [22]. Clinical control rate is defined as [(controlled + partly controlled) / total] × 100%.

### Statistical analysis

Data were analyzed using the SPSS version 23.0 (IBM Corp, Armonk, NY). Data distribution was tested for normality using the Kolmogorov–Smirnov test. Because the continuous variables were not normally distributed, they are reported as median and the first and third quartiles. The Wilcoxon signed rank test was applied for comparisons between groups. Significance was accepted at *P* < 0.05.

## Results

### Patients’ characteristics

59 patients underwent the Draf IIb procedure with LIPF technique for unilateral recurrent frontal sinus diseases by a single surgeon from 2013 to 2021. 49 patients (20 women/29 men) with a median age of 48.0 years (range 19–83 years) completed at least 12 months of follow-up (median 41.0 months, range 12–100 months) and were included in the analysis (demographic and clinical characteristics of the series are presented in Table 1). Indications included recalcitrant CRS (*n* = 32), inverted papilloma (*n* = 9) and frontal mucocele (*n* = 8). The lesion was located on the right side in 22 patients and on the left side in 27 patients. 15 patients had concomitant orbit complication (OC) with 1 case requiring trephine procedure, 3 cases were noted with allergic fungal rhinosinusitis (AFR), and 1 case had malignancy and intracranial invasion of inverted papilloma needing craniotomy.

**Table 1:** Demographic and clinical characteristics of the patients

Case no.	Side	Age (years)	Sex	Indication	Comorbidity	Concomitant operation	Follow-up (months)
1	L	50	M	IP	–	–	100
2	L	40	M	IP	–	–	92
3	R	48	M	IP	–	–	84
4	L	41	M	IP	–	–	80
5	L	52	F	IP	–	–	75
6	R	56	F	CRS	OC	–	65
7	R	56	F	Mucocele	OC	–	65
8	R	83	F	CRS	–	–	63
9	R	83	F	Mucocele	OC	–	63
10	L	38	M	CRS	–	–	61
11	L	41	M	CRS	–	–	60
12	R	33	M	Mucocele	OC	–	60
13	L	32	M	CRS	–	–	59
14	L	41	M	CRS	OC	–	57
15	R	63	F	CRS	OC	–	56
16	L	19	M	CRS	–	–	54
17	L	30	F	CRS	OC	–	53
18	R	37	M	IP	Malignancy, intracranial invasion	Craniotomy	50
19	L	26	M	Mucocele	OC	–	45
20	R	48	M	IP	–	–	45
21	L	50	M	CRS	–	–	44
22	L	46	M	CRS	–	–	44
23	L	26	M	CRS	OC	–	44
24	R	47	F	CRS	–	–	43
25	L	70	F	Mucocele	OC	–	41
26	L	61	F	CRS	–	–	40
27	R	54	F	CRS	–	–	40
28	L	51	F	CRS	–	–	39
29	L	54	M	CRS	–	–	39
30	L	50	M	Mucocele	–	–	37
31	L	61	M	CRS	OC	–	33
32	R	55	F	CRS	–	–	32
33	L	78	M	IP	–	–	30
34	L	44	F	CRS	–	–	29
35	R	30	F	Mucocele	OC	–	29
36	R	55	M	CRS	–	–	27
37	R	55	F	IP	–	–	27
38	L	29	F	CRS	AFR	–	24
39	R	46	M	CRS	–	–	24
40	R	31	M	CRS	–	–	23
41	R	31	M	CRS	OC	–	23
42	L	37	M	CRS	–	–	22
43	L	47	F	Mucocele	AFR	–	22
44	L	66	F	CRS	–	–	19
45	R	19	M	CRS	AFR, OC	Trephine	15
46	R	72	F	CRS	OC	–	14
47	R	49	M	CRS	–	–	13
48	L	65	M	CRS	–	–	12
49	R	49	M	CRS	–	–	12

*CRS* chronic rhinosinusitis, *IP* inverted papilloma, *OC* orbit complication, *AFR* allergic fungal rhinosinusitis

### Surgical outcome

The LIPF was placed in the inferior part of the nasal cavity and did not disturb the subsequent steps (Fig. 2C). All surgeries were uneventful and no perioperative complications (significant hemorrhage, orbital damage or cerebrospinal fluid leaks) were noted.

**Fig. 3:**
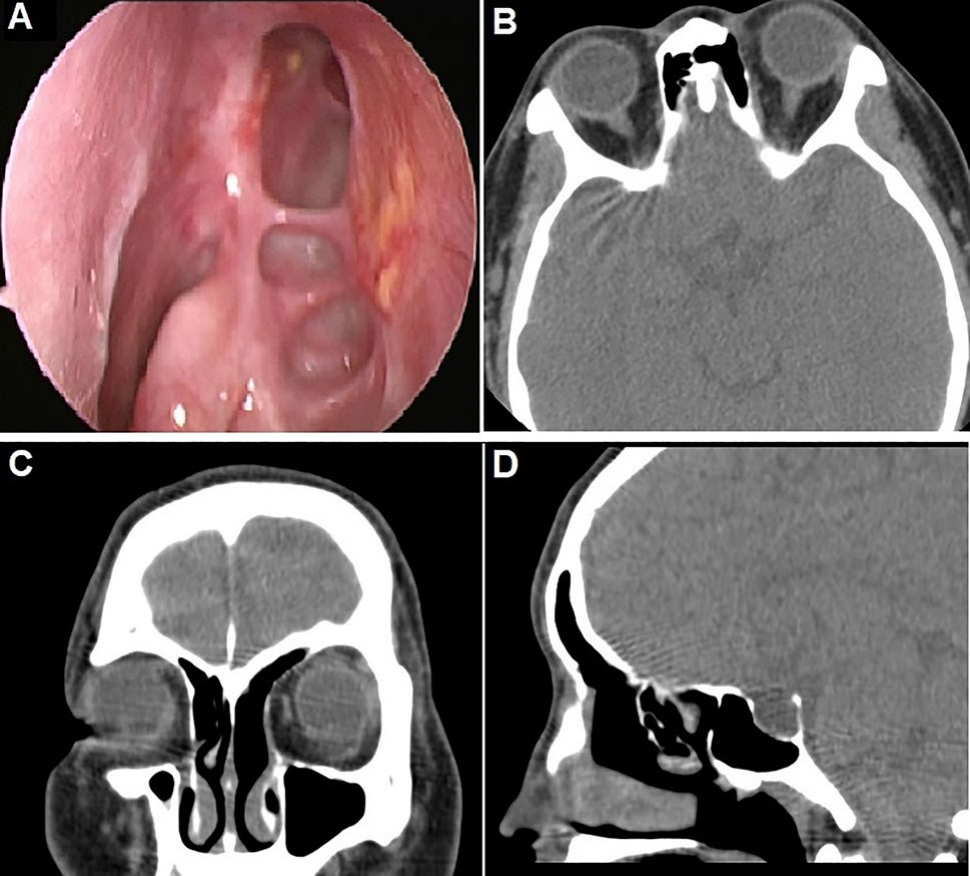
(A) Endoscopic views, (B) Axial, (C) coronal and (D) sagittal CT images showing the neo-ostium at 12 months after surgery

This LIPF can reach the frontal recess in 44 of total 49 cases (89.8%). In minority (*n* = 5, 10.2%), the length of the flap may be a little short, but it does not affect clinical outcome, because the flap will quickly grow and cover the little remained exposed bone. The underlying surface of exposed bone reached fast re-epithelialization in 3 months. Minor postoperative complications (non-obstructive synechia) were noted in four patients (8.16%). The frontal neo-ostium remained patent in all patients both on endoscopy and on CT scan (Fig. 3) at a follow-up of 12 months. Failure was noted in one patient (2.04%) for recurrence of inverted papilloma at 21 months follow-up with more than five previous surgeries.

32 patients with recalcitrant CRS were available for at least 1 year of follow-up (mean of 39 months, range 12–65 months). At the 12 months, 25 patients of them were controlled, 7 cases were partly controlled, and no one were uncontrolled. Till the current time of this article, 26 patients were controlled, 6 cases were partly controlled, and no one were uncontrolled. The facial pain and total symptom VAS scores after surgeries both decreased significantly when compared with the baseline [baseline: 5 (4, 7), 11 (6, 16) vs 3 months: 2 (1, 3), 9 (4, 11) and 12 months: 2 (1, 3), 9 (4, 11); *P* < 0.05]. The VAS scores of nasal obstruction, rhinorrhea, loss of smell and overall burden did not change significantly (*P* > 0.05). The Lund-McKay endoscopic score decreased significantly (*P* < 0.05) when compared with the baseline [baseline: 4 (3, 5) vs 3 months: 2 (2, 4) and 12 months: 2 (1, 3); *P* < 0.05]. The detailed data are shown in Table 2.

**Table 2:** VAS and Lund-Kennedy scores of patients with recalcitrant CRS

	Baseline	3 months	1 year
VAS score			
Nasal obstruction	1 (0, 2)	1 (0, 3)	1 (0, 2)
Rhinorrhea	1 (0, 3)	1 (0, 3)	1 (0, 3)
Facial pain	5 (4, 7)	2 (1, 3)*	2 (1, 3)*
Loss of smell	2 (1, 4)	2 (1, 3)	2 (1, 3)
Total symptom	11 (6, 16)	9 (4, 11)*	9 (4, 11)*
Overall burden	2 (0, 3)	2 (1, 2)	2 (1, 3)
Total endoscopic score	4 (3, 5)	2 (2, 4)*	2 (1, 3)*,^#^

*Compared with the score of baseline, *P* < 0.05

^#^Compared with the score of 3 months, *P* < 0.05

## Discussion

In this study, we prospectively report the first clinical series of a LIPF technique in Draf IIb procedures and demonstrate its clinical feasibility and efficacy associated with good outcomes in maintaining neo-ostium patency and clinical control of disease, without major complications.

The main techniques that can address advanced frontal sinus pathologies with the main concern maintaining neo-ostium patency are the Draf IIb and Draf III drillings. There is much more evidence concerning the outcome of the Draf III procedure, which is subsequently widely applied in current practice. In a 2017 systematic review and meta-analysis (level 2 evidence) of 29 articles including 1205 patients mainly indicated in chronic frontal sinusitis, mucoceles, tumors and traumas, the overall patency rate after Draf III was 90.7%, and the revision rate was 12.6% [8]. Despite studies selectively reporting on Draf IIb results in previous literature were limited, several recent studies had showed the safety and efficacy of the Draf IIb procedure. A systematic review of 26 studies by Haddad et al. in 2021 (level 2 evidence) showed that the main indication for Draf IIb was chronic frontal rhinosinusitis (61.82%), and the overall postoperative patency rate was 87.85% whether it was applied for chronic rhinosinusitis or for the other indications [10]. Patel et al. [9] also conducted a comparative cohort study (level 3 evidence) between Draf IIb and Draf III and showed no significant difference in frontal sinus patency rates, revision rates, or complications. These evidences have proved the safety and efficacy of Draf IIb comparable with Draf III, making it a valid option when a bilateral approach is not needed. In this study, all the patients underwent the Draf IIb surgery for unilateral recurrent frontal lesions and the overall outcome was favorable.

Restenosis of frontal neo-ostium is still an issue in the Draf surgery, especially in the case of unilateral Draf IIb procedure. One of the main factors associated with restenosis is related to the resection of mucosa leaving a circumferential surface of exposed drilled bone that may induce osteitis with subsequent neo-osteogenesis and stenosis [11]. Lee suggests that osteitic bone acts as an inflammatory center, initiating edema and hypertrophy of the adjacent mucosa, thus narrowing the frontal recess [23]. Mucosal flaps or free grafts can be used to cover exposed bone to speed up mucosal healing and prevent osteitis.

Covering exposed bone with grafts or flaps was proposed in Draf III with promising results [11, 15, 16, 18, 20]. Recently, several flap techniques have also been reported to improve outcomes in Draf IIb procedures. Grayson et al. [17] applied a nasoseptal flap (NSF) or free graft in 37 patients who had underwent Draf IIb for frontal sinus fractures, and all sinuses were patent on final examination at a mean follow-up of 26 months (level 4 evidence). In Fiorini et al.’s [5] report, a septoturbinal flap (STF) was used for Draf IIb procedures, and postoperative stenosis of the neo-ostium was observed in 1 of 46 patients (level 4 evidence). Khoueir et al. [19] reported on a double-flap technique using a STF and a lateral-based nasoseptal flap (LNSF) to cover the posterior and anterolateral edges of the Draf IIb neo-ostium in eight patients, and no restenosis was noted at a mean follow-up of 3 months (level 4 evidence). In Omura et al.’s study, a superior lateral anterior pedicle (SLAP) flap was applied to Draf IIb procedures in eight cases (level 4 evidence). The neo-ostium remained patent in all patients, and no complications, such as synechiae or orbital injury, were seen in any of the patients [2]. Though no statistically significant conclusion could be made in Haddad et al.’s systematic review, patency rates after Draf IIb surgery were higher when flaps or grafts were applied (93.5%) versus when they were not (86.7%) [10]. LIPF is based on the dorsum of the inferior turbinate, and mainly pedicled on branches of the facial and lateral posterior nasal arteries. In this study, the LIPF were used in all the Draf IIb surgeries, allowing fast re-epithelialization and integration with the underlying surface.

The LIPF has several advantages. First, LIPF can be used in patients who previously underwent endoscopic surgery, compared with the aforementioned flaps involving septum or middle turbinal, such as NSF, STF and LNSF. In revision surgeries, the nasal septum and middle turbinal is not always intact. Whereas the lateral nasal wall, especially the mucosa anterior to middle turbinal, is not manipulated in most cases. Second, compared with the SLAP flap raised from the inferior turbinate which is usually thick and not always suitable for maintaining the neo-ostium patency, the LIPF is thinner and more portable, and moreover, the design of LIPF can not only avoid torsion of the pedicle of flap when unfolded to cover the exposed bone, but also maintain the natural drainage outflow of frontal ostium, because the direction of mucociliary flow is orientated in LIPF. Finally, it is mini-invasive and easy to manipulate, and usually does not disturb the operative field.

In this study, our LIPF technique resulted in a high patency rate of 100% and a good clinical control rate of CRS after at 12 months. A follow-up period of at least 12 months for evaluating ostium patency was chosen for that it was demonstrated that restenosis is an ongoing process during a period of 1 year [13]. Moreover, based on EPOS 2012 [22], the short-term outcome of surgical treatment for CRS was also suggested to be evaluated for at least 1 year, which is consistent with newly published EPOS 2020 [24]. Although final size of the neo-ostium was not exactly measured and failed to compare with the intraoperative size, the ability to visualize into the frontal sinus by endoscopic assessment was able to evaluate the patency of the neo-ostium, which has been applied and validated in various previous studies [2, 9, 19, 25].

In view of this study, we rationally speculated that the LIPF can be applied to advanced frontal sinus pathologies, such as recalcitrant CRS, mucocele, benign tumor and trauma. Theoretically, there are some clinical situations where the LIPF is not suitable: unhealthy mucosa in case of severe polyposis or extensive fibrosis.

This study was level 4 evidence in accordance with the Oxford Centre for Evidence-Based Medicine Levels with some limitations. The first, there was no control group without application of flaps. The second, our patency rate of 100% and clinical control rate of CRS may be inconsistent with the recent systematic review which showed that the postoperative patency rate of was 93.5% for Draf IIb with flaps/grafts [10]. However, one previous study from Omura et al. [2] also provided an overall patency rate of 100% in their cohort, which was in line with our findings. Moreover, nasal nebulization inhalation of budesonide for CRS patients may also contribute to the excellent outcome in addition to the LIPF technique [21, 26]. The third, a larger sample size and longer follow-up might change outcomes. However, despite of these limitations, our current study suggests that the LIPF technique in the Draf IIb for unilateral recurrent frontal lesions is convenient, safe and effective.

## Conclusion

The LIPF technique was convenient and applicable to Draf IIb procedures. It could decrease the incidence of restenosis of the frontal sinus drainage pathway and increase the clinical control rate of the disease without main complications. Randomized controlled studies in homogenous groups with larger sample are needed to verify this conclusion.

## Data Availability

The data that support the findings of this study are available from the corresponding author upon reasonable request.
